# What Do We Know About Sharing Power in Co‐Production in Mental Health Research? A Systematic Review and Thematic Synthesis

**DOI:** 10.1111/hex.70014

**Published:** 2024-09-05

**Authors:** India Hopkins, Max Verlander, Lucy Clarkson, Pamela Jacobsen

**Affiliations:** ^1^ Department of Psychology University of Bath Bath UK

**Keywords:** co‐production, mental health research, power

## Abstract

**Background:**

Guidance on co‐production between researchers and people with lived experience was published in 2018 by the National Institute for Health and Care Research (NIHR) advisory group, previously known as INVOLVE. This guidance described sharing power as a key principle within co‐production. Authentic sharing of power within co‐produced mental health research does not always occur however and remains a challenge to achieve within many projects.

**Objectives:**

To explore what has been learned about the sharing of power in co‐production within mental health research since the publication of these guidelines, by synthesising qualitative literature relating to power within co‐produced mental health research.

**Methods:**

We carried out a systematic review with thematic synthesis. We searched CINHAL, Embase and PubMed databases to identify qualitative or mixed‐method studies relating to power within co‐produced mental health research. Studies were independently screened by two reviewers for inclusion and appraised using the Critical Appraisal Skills Programme tool (CASP) for qualitative research.

**Results:**

We identified nine papers that met the criteria for inclusion and were included in the synthesis. Three themes were generated: (1) Battling to share power against a more powerful system, (2) Empowerment through relationships and (3) The journey is turbulent, but it is not supposed to be smooth.

**Conclusions:**

Results highlight that power is pervasive, especially within the hierarchical systems research is often conducted within. Sharing power within co‐produced mental health research is an ongoing complex process that is not intended to be easy. Respectful trusting relationships can help facilitate power sharing. However, ultimately meaningful change needs to come from research funders, universities and NHS providers.

**Patient or Public Contribution:**

The study authors include a lived experience researcher who contributed to the review design, analysis and write‐up.

## 1 Introduction

It is increasingly acknowledged that mental health research should be conducted collaboratively with people with lived experience [1]. This type of collaboration was initially much more broadly employed in physical health research, with a slower uptake in mental health research [2]. It has been suggested this could in part be due to historical power imbalances between professionals, researchers, policy makers and mental health service users, particularly service users from racially minoritised backgrounds [3, 4]. Historically mental health research has predominately been situated within the biomedical model of research, which is likely to have contributed to these differences in power [5]. However, people who have experienced mental health problems are now increasingly involved as collaborators, thanks partly to the activism of the health equity and disability rights communities [6].

There are different degrees of involvement of people with lived experience within the design, conduct and dissemination of research. Lower‐level involvement often involves researchers engaging and consulting with people with lived experiences to ask for their perspectives [7]. This could include, for example, asking for feedback on the wording of participant information sheets. However, this limits the influence and power of people with lived experience to make a difference at a deeper level, such as being involved in setting the research question in the first place. A step up from this would include researchers and people with lived experience having ongoing partnerships and sharing in decision making, within an equal and reciprocal partnership (e.g., co‐production) [2]. Even higher‐level involvement would encompass people with lived experience taking on leadership roles, delivering research and having the dominant voice [8]. Although evidence highlighting the importance of lived experience in mental health research is increasing, research has also illustrated that services and organisations can find it difficult to negotiate moving away from the long‐standing structures within systems towards service user leadership and the sharing of power [9]. This is evident by patterns of lived experience involvement across the research cycle; for example, with involvement less likely in the analysis and writing up stage, which may reflect a reluctance of researchers to share power in these domains [10], or a lack of opportunities and training for people with lived experience to feel confident in such domains [11].

Co‐production can be viewed as a type of lived experience collaboration or involvement in research [12]. In 2018 the National Institute for Health and Care Research's (NIHR) advisory group supporting active public involvement in the NHS, public health and social care research, previously known as INVOLVE, published ‘Guidance on co‐producing a research project’ [13]. This guidance detailed that *‘*co‐producing a research project is an approach in which researchers, practitioners and the public work together, sharing power and responsibility from the start to the end of the project, including the generation of knowledge’. The five key principles of co‐production as described in this guidance include: (1) Sharing power, (2) Including all perspectives and skills, (3) Respecting and valuing the knowledge of everyone, (4) Reciprocity and mutuality and (5) Understanding each other. It is vital these principles are employed during co‐production; however, this does not always occur. Consequently, research claiming to have been co‐produced and has not directly acknowledged or supported power sharing throughout the research process has been criticised [2, 14]. Conversely, the NIHR has highlighted some helpful examples of co‐produced research that illustrate how the key principles of co‐production have been practically applied [15, 16].

To promote ethically sound co‐production, relational dynamics need to be considered [17]. In terms of co‐production in mental health research power and relational dynamics, as well as how these may impact the process of co‐production are especially important to acknowledge, as people with lived experience of mental health problems continue to face stigma and marginalisation in society [18]. Additionally, these power dynamics can act as a significant barrier to the involvement of people with lived experience [19]. In this case, power can be described as the level of control someone has over an activity and can comprise final decision making, as well as determining the types of experience and knowledge that are thought to be valid [4]. Therefore, considerable power‐related barriers remain and the lack of acknowledgement of these in some studies can impede meaningful co‐production [3, 20].

In conducting this review, our understanding of power was influenced by and anchored to various theories. The first being Michel Foucault's relational perspective on power [21, 22]. From this we understand power not as something someone merely has, owns or that simply emerges from powerful institutions, but rather something that develops and is present in all relationships. Within this concept, it is also important to note power and knowledge are inevitably linked. Relating to this and another way in which one can understand power is through the concept of epistemic injustice [23], which is a form of discrimination that occurs when people from marginalised groups are seen as unreliable creators of knowledge and are often excluded from the process of knowledge and meaning creation [24]. Historically, people with lived experience of mental health problems have been deemed as unreliable knowers or the knowledge of their own problems has been discredited [25]. This continues today and notably impacts people from racially minoritised backgrounds who have experienced mental health problems [26]. Studies have highlighted ways in which epistemic injustice within mental health research can be addressed. Such as, appropriately embedding and employing lived experience roles within research institutions, as well as creating new spaces and structures where people with lived experience can pursue their own research priorities and where alternative forms of knowledge are accepted and appreciated [27]. Additionally, the ‘Social Ecology of Power’ is a conceptual framework that situates power within a wider interconnected system and allows for the exploration of how power operates within and across the individual, interpersonal and structural levels [28].

The evidence base exploring the involvement of people with lived experience of mental health problems in research has increased over recent years. A previous systematic review synthesised numerous frameworks that support patient and public involvement in health research, such as the Public Involvement Impact Assessment Framework (PiiAF) [29]. A more recent scoping review explored the barriers, facilitators and outcomes of co‐production in psychosis research [30]. Findings from this review highlighted power imbalances as a common barrier and effective communication as a facilitator of co‐production. Additionally, a critical review investigated how power was addressed in relation to service user involvement in mental health professions education [31]. However, there is a gap in the literature regarding the sharing of power within co‐produced mental health research, specifically after the publication of the 2018 INVOLVE ‘Guidance on co‐producing a research project’. We aimed to ground our understanding of power sharing in co‐produced mental health research, in the lived experiences of people with mental health problems who have been involved in this type of research. We chose to focus on developments after the 2018 INVOLVE guidance was published as this was a watershed moment in the development of co‐production in mental health research in the United Kingdom and may have led to increased focus on this within subsequent research projects.

The primary question of this review was ‘What have we learned about the sharing of power in co‐production within mental health research since the 2018 INVOLVE “Guidance on co‐producing a research project” was published?’.

## 2 Methods

We pre‐registered the protocol for the review on the Open Science Framework on 20 December 2023, available here: https://osf.io/tpkmz. This review is reported in line with ENTREQ guidelines for qualitative syntheses [32] and PRISMA guidelines [33]. The ‘PICo’ framework for developing qualitative research questions for systematic reviews was used to develop the research question and search strategy [34]. In line with this framework, the *population* referred to studies that employed co‐production or involved people with lived experience in all stages of the research process. The *interest* referred to power and the *context* was within mental health research, specifically after the publication of the 2018 INVOLVE ‘Guidance on co‐producing a research project’.

### 2.1 Search Strategy and Information Sources

A comprehensive search strategy was developed, including co‐production, mental health and power (see Supporting Information Material for search terms). The databases searched for this review were CINHAL, Embase and PubMed. The searches were run on 2 January 2024.

### 2.2 Inclusion and Exclusion Criteria

The inclusion and exclusion criteria were developed to identify studies with qualitative data, referring to co‐production and power in the context of mental health research. The specific inclusion and exclusion criteria can be seen in Table 1.

**Table 1:** Summary of inclusion and exclusion criteria.

Inclusion	Exclusion
Qualitative or mixed‐methods studies including qualitative data	Quantitative only studies
Studies that have been conducted in the United Kingdom *(The INVOLVE ‘Guidance on co‐producing a research project’ was developed in the United Kingdom by a group that supports public involvement in the NHS. Therefore, it was felt UK studies were more likely to have heard of these guidelines and subsequently refer to them, hence the focus on UK studies only)	Editorials, reviews, commentaries or study protocols
Studies that were published from 2018 onwards *(This criterion was anchored to 2018 as this was the year the INVOLVE ‘Guidance on co‐producing a research project’ was published)	Intervention studies where qualitative data only explores the experience of that intervention and does not relate it to the process of co‐production or the sharing of power within this process
Studies that were co‐produced or involved people with lived experience of mental health problems in all stages of the study, not just as participants but as collaborators throughout the research process *(A formal diagnosis of mental health problems was not a requirement for this criterion, studies that involved people with self‐reported mental health problems were also included)	Studies that were not published in a peer‐reviewed journal
Studies that report qualitative data of relevance to co‐production and power	

### 2.3 Screening and Data Extraction

We used the review software programme Covidence to assist in conducting the review. During the first stage of screening, two reviewers independently screened all articles by title and abstract against the inclusion and exclusion criteria. Following this, potentially eligible articles were put through to the second stage of screening. During this stage, two reviewers independently reviewed the full‐text papers. Any conflicts between reviewers' decisions were resolved through further discussion, followed by consultation with the senior author (P.J.) if required to reach a consensus.

We developed a standardised data extraction template. In line with the aims of the review, four types of qualitative data relating to co‐production and power were extracted: (1) Key concepts, themes or patterns identified in each study, sometimes referred to as first order interpretations; (2) Data extracts related to the key concepts, themes or patterns for example, sample quotes; (3) Author explanations of the key concepts, themes or patterns, sometimes referred to as second order interpretations and (4) Recommendations or conclusions made by authors.

### 2.4 Quality Assessment

The Critical Appraisal Skills Programme tool (CASP) for qualitative research was used to assess the quality of the papers that met the inclusion criteria [35]. It comprises 10 criteria, including two questions related to screening and eight related to the quality of the studies. This tool was chosen as it examines the quality of studies across multiple areas, allowing for a comprehensive assessment of quality. Two reviewers independently completed the quality assessment of all studies, with any conflicts resolved by discussion to reach a consensus.

### 2.5 Data Analysis

Thematic synthesis was used to analyse the data [36] and was chosen as it allows existing evidence to be brought together and patterns within the data to be identified. A qualitative synthesis was chosen as the research team felt it was best placed to answer the more open‐ended research question and would allow more in‐depth insights into the topic in comparison to a quantitative synthesis. An inductive approach to analysis was employed as it allowed the researcher to remain close to the experiences of people involved in each study, in addition to ensuring the final themes were data‐driven. It is also important to acknowledge that during this process the researchers' understanding of power was anchored to the relational perspective on power [21, 22], the concept of epistemic injustice [23] and the ‘Social Ecology of Power’ framework [28]. These power‐related theories and frameworks helped inform the researchers' interpretation of how power operates in co‐produced mental health research, hence helping to guide and structure the synthesis.

Data analysis included three stages: (1) Coding of text line by line, (2) Development of descriptive themes and (3) Development of analytical themes. During line‐by‐line coding of qualitative data, where appropriate, codes were copied across studies to facilitate the translation of concepts across studies. Coding was done by the first author (I.H.) and discussed with a member of the research team (L.C.) who was a person with lived experience. Descriptive and analytical themes were then generated by I.H. and L.C. and discussed with the wider research team. Descriptive themes stay close to the data and aim to summarise multiple codes by grouping similar ones together. Analytical themes move beyond the data, they aim to describe the concepts or patterns of meaning that underly the descriptive themes and how these link to the research question.

### 2.6 Research Team and Reflexivity

The core analysis team consisted of I.H. (trainee clinical psychologist), L.C. (lived experience researcher) and P.J. (clinical psychology academic). The analysis team all identified as female, White Irish or British and were in the 20s−40s age range. They also all had experience of being involved in co‐produced mental health research. L.C.'s perspectives on the analysis were critical to facilitate reflection on the role of the lived experience researcher and the sharing of power in co‐produced mental health research. L.C. provided commentary on the descriptive and analytical themes and reviewed the final draft of this paper.

Notes were kept by the first author to reflect on what they were bringing to the analysis regarding their previous experiences and preexisting assumptions. This promoted self‐reflexivity and helped them to remain aware of their own biases. Regular research supervision with the senior author (P.J.) was also used to reflect on the codes and themes generated.

## 3 Results

### 3.1 Identification of Studies

**Figure 1:**
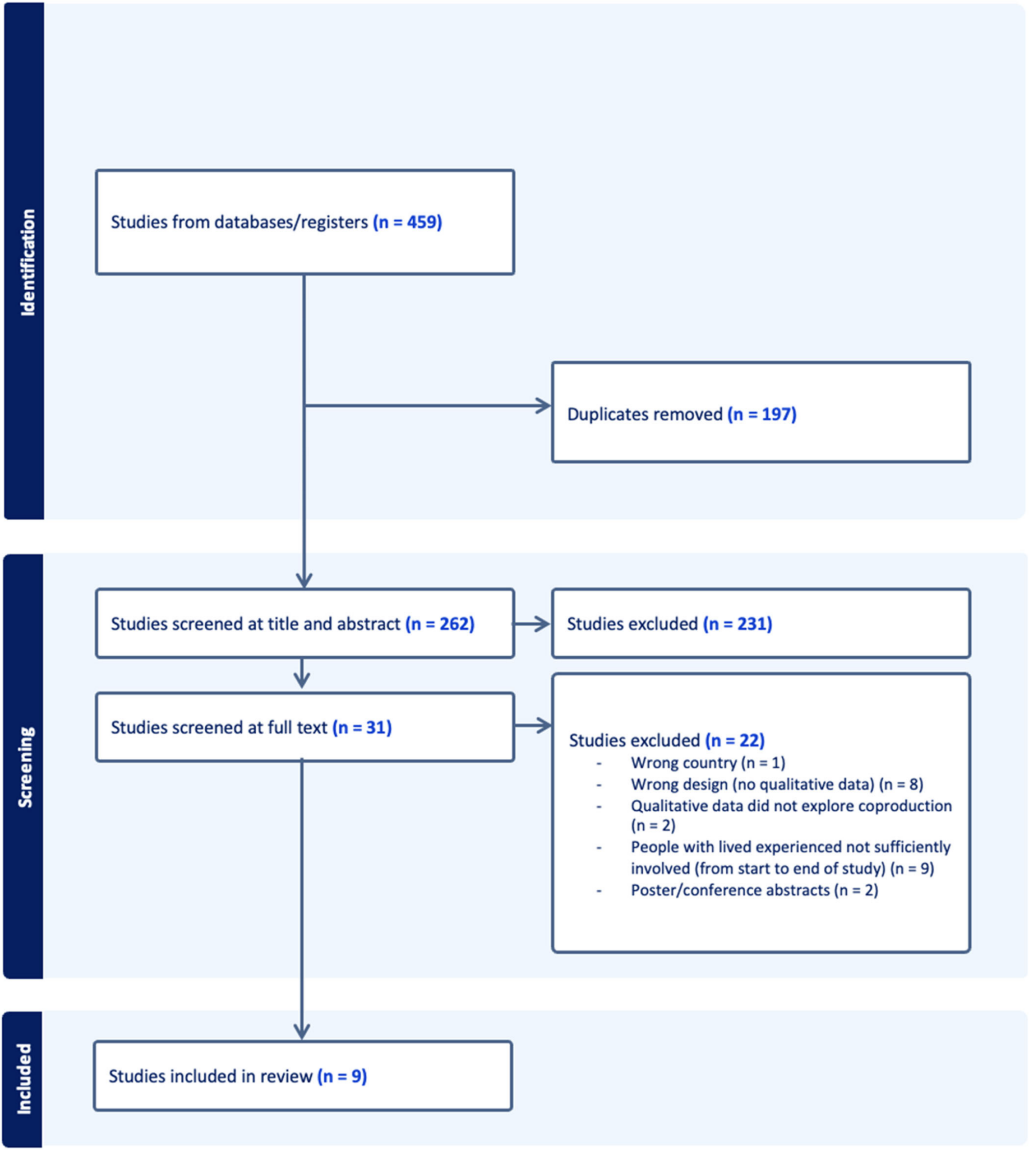
PRISMA diagram of the screening process.

After removing duplicates, a total of 262 papers were identified for screening. Following this, 231 papers were excluded at the title and abstract review stage. A total of 31 full texts were reviewed. Of these, nine were included in the synthesis. See Figure 1 for the PRISMA diagram.

### 3.2 Study Characteristics

The nine studies included in synthesis, see Table 2, were conducted in the United Kingdom and none were mixed methods. Most papers used semi‐structured interviews as part of their data collection method (*n* = 6), others used focus groups (*n* = 1), reflective accounts (*n* = 2), discussion forums (*n* = 1), and data from documents (*n* = 2), with three papers using a combination of these methods. Most papers employed thematic analysis (*n* = 4), with others referring to the generation of themes or categories, but not stating they explicitly followed a thematic analysis approach (*n* = 3). A total of three papers referred to the INVOLVE ‘Guidance on co‐producing a research project’.

**Table 2:** Descriptions of study characteristics—Some descriptions taken verbatim from original papers.

References	Research question/aim	Sample	Method of data collection	Analysis method	Does the study refer to INVOLVE guidelines
PARTNERS2 Writing Collective [37]	To explore the approach to integrating lived experience expertise in the PARTNERS2 research programme	21; 11 members of the three Lived Experience Advisory Panels (LEAPs), eight researchers (some of whom were service user researchers), the PARTNERS2 PPI lead and the PPI co‐ordinator/peer researcher	Reflective accounts from team members	Generation of ideas of key messages, which consisted of five overarching themes using a pragmatic inductive approach	Yes—authors compared their experiences of co‐production against the INVOLVE guidelines
Budge et al. [38]	To explore participants' experiences of the democratic processes of a peer‐led organisation	16; Participants were part of one of three groups 1) Participants who attend The Bridge Collective 2) Bridge Collective paid staff, who also hold participant roles 3) External stakeholders for example, professionals who collaborated with The Bridge Collective in facilitating reflective spaces, supporting participants' involvement, organising talks and conferences, and monitoring The Bridge Collective's funding	Focus groups, semi‐structured interviews and three community documents	Thematic analysis	No
Dewa et al. [39]	To explore young people's perceptions on the feasibility of using technology to detect mental health deterioration	7; All were young people (aged 18−25) and were appointed to the Young People's Advisory Group (YPAG), two male, five White‐British, one British‐Asian, one Black‐British, all had lived experience of mental health difficulties, including depression, anxiety disorder, bipolar disorder, anorexia nervosa, psychosis, substance misuse and personality disorder. Three young people became co‐researchers	Semi‐structured interviews	A co‐produced thematic coding framework and thematic map	Yes—study was evaluated against the INVOLVE guidelines
Evans and Papoulias [40]	To explore the development of PPI within a London‐based mental health biomedical research centre over a period of 10 years	16; Six men, 10 women, seven were non‐service user staff in academic, clinical academic and nonacademic management roles, four were salaried service user researchers/academics, (researchers with lived experience of mental distress who were staff in the university), five were service user advisors (service users working in an advisory capacity who were not staff in the university)	52 organisational documents and semi‐structured interviews	Thematic analysis	No
King and Gillard [41]	To explore the impacts of a participatory‐informed approach to coproducing mental health research on overcoming barriers to knowledge co‐production	13; The evaluation was led by an experienced, university‐based survivor researcher, Colin (Black British man) with the support and guidance of a health services researcher, Steve (White British man), 11 participants were recruited as part of the evaluation, seven from the local community, four from wider networks, eight women, three men, four Black or Black British, four White or White British, two Asian or Asian British and one Other Ethnic Group	First‐person reflective narratives from authors and feedback from the co‐researcher team on their experiences of undertaking the project	Independently written first‐person accounts of authors' experiences of the project, which were co‐edited and organised under subheadings	No
McGeown et al. [42]	When working with people who have experienced multiple traumas, how do co‐production approaches need to be developed to ensure safe, collaborative and effective working relationships?	43; All were women, 29 with lived experience of trauma, four researchers, two academic GPs, four GP trainees, four One25 staff members (a charity that supports some of the most marginalised women in Bristol)	Reflective notes, observations of meetings, semi‐structured interviews with people involved in the project and reflective group discussions on the team's experiences	Open coding of observation and reflective notes using an inductive approach. Identification of emergent categories relating closely to the six principles of trauma‐informed approaches. A framework approach was adopted in subsequent analysis	Yes—compared principles to trauma‐informed principles
Thomson et al. [43]	1. To explore how a diverse range of children and young people (CYP) could become interested and engaged in the research process 2. To develop a model of meaningful engagement of CYP in research 3. To codevelop a programme of meaningful research ‘with’ CYP, rather than ‘for’ them	20; Young people (aged 21−25)	Discussion forums	Thematic analysis	No
West et al. [44]	To explore the experiences of a person with dementia, their family and mental health staff, involved in co‐producing a course about ‘living well’ with dementia, within a mental health Recovery College	3; One person with dementia, one family member of person with dementia, two staff members	Semi‐structured interviews	Thematic analysis	No
Worsley et al. [45]	To evaluate the process of coproducing a mental health‐related research proposal suitable for funding through a national health research funding body	The working group initially comprised four research professionals and 11 service users, carers and members of the public. Only five public advisors sustained involvement throughout the entire process, becoming co‐applicants on the research proposal. Reflections from three applied health research professionals (two men, one woman) and three public advisors (two men, one woman)	Semi‐structured interviews	Thematic analysis	No

### 3.3 Quality Assessment

The quality of the papers included in this review varied somewhat, specifically in the description of the recruitment strategies used, as well as the consideration of the relationship between the researcher and participants. However, all papers clearly stated their aims, which indicated a qualitative methodology was appropriate, and provided justifications for the use of this methodology. Additionally, for all papers, the discussion of findings was thoroughly considered, and the research was valuable. See Table 3 for the ratings of each paper against the CASP checklist.

**Table 3:** Quality assessment using CASP checklist.

	CASP questions									
References	1. Was there a clear statement of the aims of the research?	2. Is a qualitative methodology appropriate?	3. Was the research design appropriate to address the aims of the research?	4. Was the recruitment strategy appropriate to the aims of the research?	5. Was the data collected in a way that addressed the research issue?	6. Has the relationship between the researcher and participants been adequately considered?	7. Have ethical issues been taken into consideration?	8. Was the data analysis sufficiently rigorous?	9. Is there a clear statement of findings?	10. How valuable is the research?
PARTNERS2 Writing Collective [37]	Yes	Yes	Yes	Yes	Yes	Yes	Yes	Yes	Yes	Valuable
Budge et al. [38]	Yes	Yes	Yes	Yes	Yes	Yes	Yes	Yes	Yes	Valuable
Dewa et al. [39]	Yes	Yes	Yes	Yes	Yes	Yes	Yes	Yes	Yes	Valuable
Evans and Papoulias [40]	Yes	Yes	Yes	Yes	Yes	No	Yes	Yes	Yes	Valuable
King and Gillard [41]	Yes	Yes	Yes	Yes	Yes	Yes	Can't tell	Yes	Yes	Valuable
McGeown et al. [42]	Yes	Yes	Yes	Can't tell	Yes	Yes	Yes	Yes	Yes	Valuable
Thomson et al. [43]	Yes	Yes	Yes	Yes	Yes	No	Yes	Yes	Yes	Valuable
West et al. [44]	Yes	Yes	Yes	Yes	Yes	Yes	Yes	Yes	Yes	Valuable
Worsley et al. [45]	Yes	Yes	Yes	Yes	Yes	No	Yes	Yes	Yes	Valuable

### 3.4 Synthesis of Results

**Figure 2:**
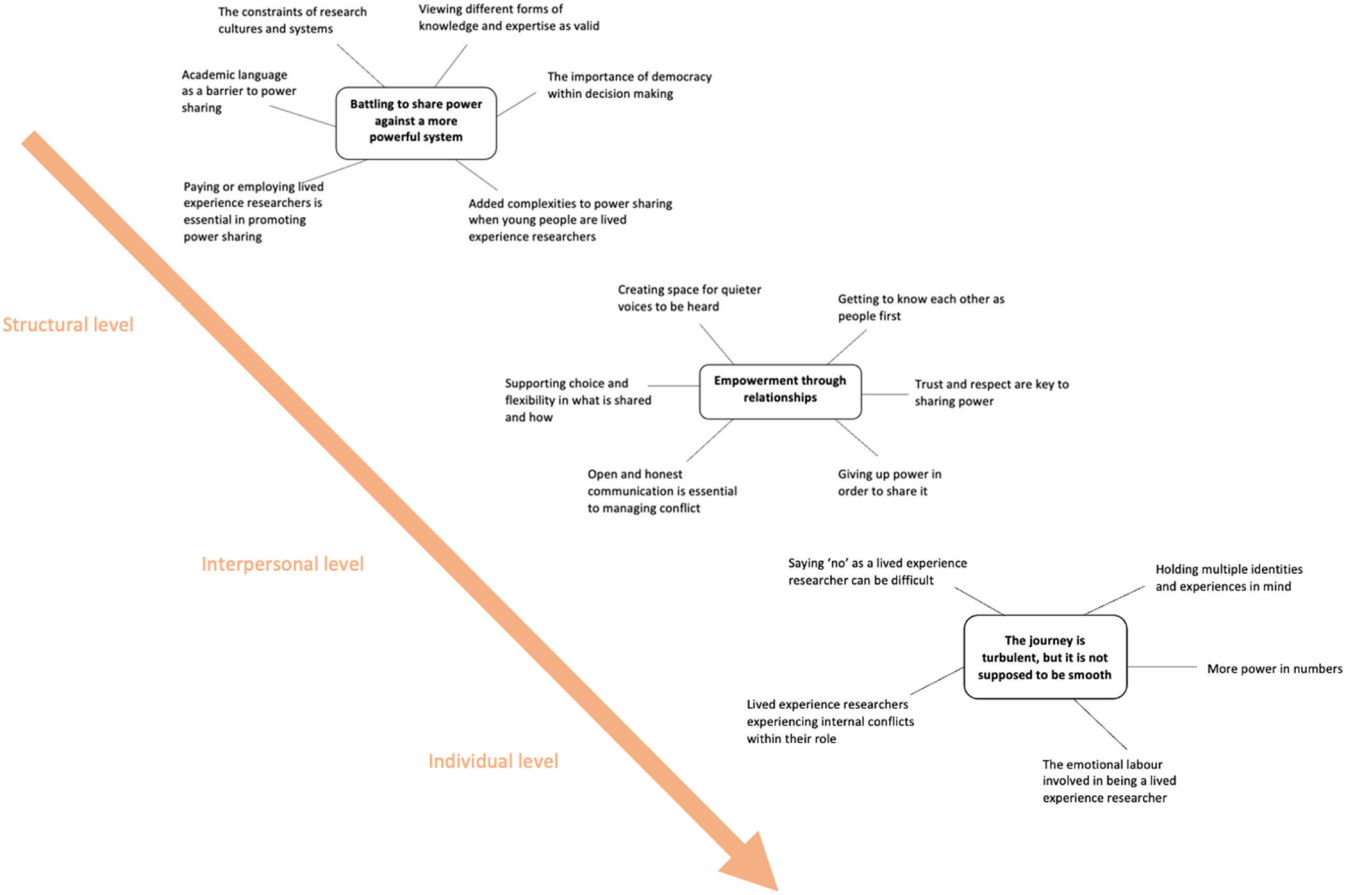
Thematic map.

Three analytical themes were created within the synthesis: (1) Battling to share power against a more powerful system, (2) Empowerment through relationships and (3) The journey is turbulent, but it is not supposed to be smooth. These illustrate how power can present and operate within structural, interpersonal and individual levels in terms of co‐produced mental health research. It is important to note that while these themes are initially described as relating to the three different levels separately, they are also inextricably linked, with each level sharing various layers of interconnectedness. See Figure 2 for a thematic map, which outlines the analytical and descriptive themes generated.

#### 3.4.1 Theme 1: Battling to Share Power Against a More Powerful System

This theme fits with how power can present and operate at a structural level regarding co‐production. Research is often conducted within the confines of academic and mental health systems, which are inherently hierarchical, and as a result, are seen to hinder true power sharing. It was acknowledged that the entrenched research cultures contained within Higher Education Institutions (HEI) and NHS services do not support the authentic sharing of power between professional and lived experience researchers.

Individual ‘breakthroughs’ in reciprocity and relationship building can feel short‐lived…. We have no shortage of ideas or goodwill but changing research cultures fundamentally remains a huge challenge.

PARTNERS2 Writing Collective [37], p. 7, quotation from participant

Overall service users were expected to fit in rather than transform the cultures and priorities of the organisation*.*

Evans and Papoulias [40], p. 2

Despite all the rhetoric around NIHR for public engagement, and you get to write about it on one of the boxes on the forms, I think the system's commitment to an authentic messy public engagement is not there yet.

Worsley et al. [45], p. 6, quotation from participant

The use of academic language perpetuated how power operated at a structural level, acting as a barrier to authentic power sharing. It was acknowledged that professional researchers often employ this type of language without fully appreciating the barrier it can construct.

Although we thought that we were pretty good at communicating ideas without using academic type language… You're never aware that you slip into it subconsciously all the time because it's a professionalised language.

Worsley et al. [45], p. 6, quotation from participant

It's really changed… how we communicate with each other……'cos we're just so used to talking to each other in this coded language.

Evans and Papoulias [40], p. 8, quotation from participant

I am haunted by the fluency of the research language, the depth of the coded language used and the fear of the challenge of carrying out the research into a mental health project I know nothing about.

King and Gillard [41], p. 4, quotation from participant

Within this theme, the importance of employing lived experience researchers was highlighted as a way in which systems could support power sharing. Advocating to compensate lived experience researchers for their valuable contributions acknowledges their expertise and can be a step towards promoting equitable participation.

If you want to really meaningfully involve people with lived experience with mental health or any other experience actually you have to employ them.

Worsley et al. [45], p. 7, quotation from participant

I think payment should be given if they're wanting your expertise and calling you an ‘expert by experience’.

Worsley et al. [45], p. 3, quotation from participant

#### 3.4.2 Theme 2: Empowerment Through Relationships

This theme fits with how power can present and operate at an interpersonal level regarding co‐production. Getting to know each other as people and fostering trusting, respectful relationships between professional and lived experience researchers was seen to be a vital component in empowering people and promoting power sharing.

feeling respected by people that have quite a lot of power [is] like the reverse…[of my experience of services]…it's been helpful on a personal level.

Evans and Papoulias [40], p. 8, quotation from participant

We generated ideas and spurred each other on. We also made alliances and friendships … where we supported each other within and outside meetings in regard to certain points of work or emotional support. This was important for the rapport and cohesion of the team in sustaining motivation and morale.

King and Gillard [41], p. 5, quotation from participant

It's been really nice to work alongside in that more partnership way, with someone with dementia and a carer. And I think that just implicitly brings that message home about people are still people, and we all need that don't we? And to be reminded of that.

West et al. [44], p. 3, quotation from participant

Since I've been in with this research and the people, I've felt as though I can open up and I could talk. They know more about me and that's given me trust to be able to talk.

Worsley et al. [45], p. 4, quotation from participant

There appeared to be consensus that through these relationships lived experience researchers should be supported to choose what they want to share in terms of their lived experience and how they want to share this. Recognising the importance of honouring individual autonomy and preferences during research and promoting a sense of agency and ownership.

I learnt to develop respect for the differences that existed and for some that might mean more disclosure than others.

PARTNERS2 Writing Collective [37], p. 4, quotation from participant

Participants valued being able to share power through being involved in a way they chose, allowing them to relax and be themselves*.*

Budge et al. [38], p. 11

‘What's going to be helpful to you emotionally as well as the project?’ and you don't have to divulge everything to make change! You don't have to prove that, ‘I've been through this and this and this’.

McGeown et al. [42], p. 10, quotation from participant

Additionally, creating space for quieter voices within the process of co‐production was seen as a way in which supportive, trusting relationships can help facilitate power sharing.

The meetings have felt inclusive and very well facilitated to try and give as many people as possible a chance to speak and contribute, despite some of the challenges that some of those people might face to do with confidence or anxiety.

Budge et al. [38], p. 12, quotation from participant

just because somebody has not said something it does not mean they have not got an opinion, but it's how, how is that opinion going to be heard, you know?, operating from a democratic point of view in the meetings.

Budge et al. [38], p. 12, quotation from participant

#### 3.4.3 Theme 3: The Journey Is Turbulent, But It Is Not Supposed to be Smooth

This theme fits with how power can present and operate at an individual level regarding co‐production and highlights the intricate dynamics involved in attempting to share power. The process of sharing power at an individual level should not necessarily be easy; it is and should be complex and cause disruption and admitting this is important. Acknowledging the emotional labour entangled with the role of lived experience researcher and the recognition of this labour is critical if genuine sharing of power is to occur.

I am not an academic. I am a mother wading my way through the mental health system, so it's an emotional journey too.

PARTNERS2 Writing Collective [37], p. 4, quotation from participant

Though experts by experience have a lot to offer, offering knowledge based on that experience can be potentially triggering.

Thompson et al. 2022, p. 4

The internal conflicts lived experience researchers can face in terms of their role was another example of how power can operate at this individual level. Lived experience researchers often felt a pull between wanting to do what was best for the research; however, also understood the consequences of what some of those decisions might mean for other people with lived experience.

We were trying to decide on which outcome questionnaires participants would be completing. I felt very conflicted. One of the favoured questionnaires asked about work and claiming benefits. I could see from a researcher point of view why this choice made sense. But I felt I was betraying service users because recording outcomes about benefits and work‐related issues can cause stress and raise fears that people might lose benefits.

PARTNERS2 Writing Collective [37], p. 4, quotation from participant

Additionally holding multiple identities and perspectives in mind was an important element of the process of power sharing at this individual level. People are complex and navigating intersecting identities and experiences within the research context requires a nuanced understanding and appreciation of diversity.

I joined a PARTNERS2 LEAP as someone who has used mental health services, but I also brought other identities, some more visible than others: carer for my mum, ethnic minority background, gay man, Muslim.

PARTNERS2 Writing Collective [37], p. 6, quotation from participant

our co‐researchers were welcomed into the university and made to feel comfortable enough that they could bring the whole of their self to the evaluation process, rather than having either to perform as a researcher to be accepted or to conform to a prescribed ‘service user’ identity.

King and Gillard [41], p. 7

There was also a mention that those who take on the role of professional researcher may have experienced mental health problems. However, this was not always named or acknowledged within the process of co‐production, contributing to a divide between professional and lived experience researchers and by extension a barrier to authentic power sharing.

Being labelled as a ‘normal’ researcher negated my own insights from actually having mental health experiences ‐ simply because it wasn't in my job title or expected of me. This made me feel uneasy in the sense of being labelled as an outsider in our LEAP, by people in the group stereotypically labelled as outsiders themselves…. None of this was ever discussed openly.

PARTNERS2 Writing Collective [37], p. 6, quotation from participant

I assumed a role of academic researcher and felt both connection and disconnection to other people around me [because of my own mental health experiences]. There was so much discussion and work around PPI in the project that this position of being ‘Inside Out’ felt very strange.

PARTNERS2 Writing Collective [37], p. 5, quotation from participant

## 4 Discussion

This systematic review aimed to synthesise qualitative data relating to sharing power within co‐produced mental health research. Nine papers met the inclusion criteria and were synthesised using thematic synthesis. All papers were qualitative studies from the UK, with participants varying in age from 18+, including professional researchers, clinicians and people with lived experience of various mental health problems, such as depression and anxiety, as well as other factors that can impact mental health, such as trauma and dementia. Three analytical themes were created.

Findings highlighted the enduring struggle those involved in research face when attempting to share power within the confines of systems not set up to encourage genuine power sharing. The available data highlights ongoing challenges posed by systemic and structural power differentials in mental health research [46, 47]. Additionally, there exists a perspective conveying scepticism about the ability of co‐production to facilitate genuine collaboration and sharing of power [48]. This perspective suggests true power sharing is impossible as power imbalances are deeply rooted in the systems we operate within [49]. It has been argued that genuine sharing of power will not occur unless profound attention is placed on the underlying epistemic injustices continuing to permeate through mental health systems and research [50]. Paying particular attention to entrenched power hierarchies surrounding race and the legacy of colonisation is an essential part of this work [51]. In addition, organisational norms that do not encourage the acknowledgement and discussion of power differences need to be challenged [20]. Prioritising the inclusion of lived experience input in decision‐making processes from the outset, providing adequate training and resources to both professional and lived experience researchers, establishing clear policies and frameworks mandating and facilitating shared governance and transparent compensation for all contributors are examples of top‐down changes that can be implemented by academic, clinical and research systems to help move towards authentic power sharing.

Previous literature suggests power is inherently relational and while power can be enacted through relationships it can also be shared and redistributed through them [52]. This aligns with the findings from our study, which highlight that supportive, trusting and respectful relationships can empower people during the process of co‐production, by extension helping to facilitate the sharing of power. Research has proposed that trust is the foundation of navigating power relations and that there is no endpoint where complete neutrality in terms of power imbalances is achieved. Rather, there is continued renegotiation of power relations and consequently continued renegotiation of power sharing [53]. Ultimately the sharing of power within relationships is not static and should be seen as an ongoing process that requires commitment and is navigated as a collective. More research into practically how this can be navigated is needed.

Findings suggested that co‐production and the journey of sharing power within this process by its nature should not be smooth, it should be turbulent and cause challenges. Existing literature has highlighted the numerous challenges that can occur within the process of co‐production, such as ensuring equitable and meaningful participation from all those involved and navigating conflict [54, 55]. The importance of holding multiple identities and perspectives in mind is a compelling element within this theme and can be linked to the complexities of sharing power in co‐produced mental health research. Previous studies have described the significance of comprehending complex intersectional identities if we are to understand the intersectional issues of power and privilege linked to being involved in co‐produced mental health research and sharing power within this context [3]. Again, it is vital to acknowledge here the systemic racism experienced by racially minoritised communities and how this upholds institutional power and often blocks genuine power sharing from occurring [56]. Our findings highlighted not only the intersectional identities professional researchers hold in terms of being viewed as professionals but also having themselves experienced mental health problems. Existing research points to the importance of keeping this in mind, while also acknowledging the additional layers of emotional labour professional researchers from racially minoritised backgrounds face in terms of their intersectional identities [3].

The ways in which power can present and operate within different domains or levels are heavily interconnected, as illustrated by the ‘Social Ecology of Power’ framework [28]. Consequently, the sharing of power within co‐production at these different levels and the themes generated from our study are immeasurably linked. For example, there appears to be an expectation within co‐produced mental health research that building trust can occur within systems that have been historically constructed to create and maintain power hierarchies [57]. However, of course, there remains an understandable mistrust of these systems from people with lived experience of mental health problems [58]. Therefore, asking these individuals to trust professional researchers who work within the very systems that have historically discriminated against them is counterintuitive. Rather professional researchers need to do the work of actively recognising and challenging their own privileges and biases and promote a constant practice of critical reflection and dialogue to challenge structural hierarchies and support power sharing [59]. We know that challenging entrenched hierarchies and power imbalances within the realm of co‐produced mental health research were never going to be a smooth endeavour. Genuine power sharing involves a fundamental shift in the dynamics of traditional research, which will not sit comfortably or be met with enthusiasm from everyone. However, feeling comfortable will not bring about change regarding sharing power in co‐produced mental health research, and professional researchers, specifically those from White backgrounds, need to use their privilege to help promote change within systems.

It is important to think about how co‐production is reflected in mental health contexts outside of the UK, as there has been significant work regarding power sharing in this area in other countries, such as Australia, which have provided more practical suggestions on how power imbalances can be addressed. For example, lived experience researchers setting meeting agendas and deciding the amount of time spent on certain topics or activities, having discretionary funds within budgets to support the group's objectives for example, include money for training within the research budget, and setting aside time to regularly review how the partnership between lived experience and professional researchers is going [60]. As the field of co‐produced research evolves it is important to think about continuing to develop these practical suggestions, as the INVOLVE guidance was designed to clarify the key principles of co‐producing a research project rather than provide a fixed set of tools or techniques on how exactly to conduct co‐produced research. Further exploration of practical tools and templates for project planning, conflict resolution and evaluation of co‐production processes could further support effective implementation of the key principles set out in the INVOLVE guidance.

## 5 Strengths and Limitations

A key strength of the review was having a person with lived experience as a core member of the research team, which helped to keep the lived experience perspective at the centre of the work. We also wrote a detailed protocol in advance and pre‐registered on a publicly accessible platform (Open Science Framework) hence enhancing transparency and reproducibility of the work.

The goal of the review was to explore how the research landscape relating to sharing power within co‐produced mental health research had changed since the publication of the INVOLVE ‘Guidance on co‐producing a research project’ in 2018. However, we are aware that a publishing lag exists and therefore the moment the guidelines were published would not have resulted in an immediate impact on the landscape. Therefore, the findings from this study are somewhat limited in terms of describing this landscape. This review focused only on UK studies and excluded quantitative studies that may have contained survey data, both of which can be seen as further limitations.

## 6 Conclusion

Findings highlight that navigating sharing power is an ongoing complex process within co‐produced mental health research and that taking time to develop supportive and respectful relationships can help facilitate power sharing. However, we know that power is pervasive and despite many researchers acknowledging the presence of power and engaging with processes to help redistribute and share power, it is at its core a systemic issue. Therefore, requires a largely systemic solution. Meaningful change regarding sharing power in co‐produced mental health research needs to come from the top down; for example, from research funders, universities and NHS providers. The journey to move towards genuine power sharing will not be smooth. However, as we described previously, it is not intended to be, as the very purpose of including a range of perspectives in research is to provide diverse viewpoints and a challenge to the status quo.

## Author Contributions

**India Hopkins:** conceptualisation, writing–original draft, formal analysis, methodology, visualisation, writing–review and editing. **Max Verlander:** writing–review and editing, validation. **Lucy Clarkson:** writing–review and editing, formal analysis. **Pamela Jacobsen:** writing–review and editing, supervision.

## Conflicts of Interest

The authors declare no conflicts of interest.

## Supporting information

Supporting information.

## Data Availability

Data sharing is not applicable to this article as no new data was created or analysed in this study.
