# Supplementary material for: What Do We Know About Sharing Power in Co‐Production in Mental Health Research? A Systematic Review and Thematic Synthesis
Source: Health Expect. 2024 Sep 5;27(5):e70014. doi: 10.1111/hex.70014 (PMC11375733; doi:10.1111/hex.70014)
Supplement: Supplementary file 1 — Supporting information. [file HEX-27-e70014-s001.docx]

**Supplementary material: Search terms for literature review**

(“co production” OR “co design” OR “co creation” OR “co delivery” OR “co innovation” OR “co evaluation” OR coproduction OR codesign OR cocreation OR codelivery OR coinnovation OR coevaluation OR “patient and public involvement” OR “partnership working” OR “partnership research” OR “peer research” OR involvement OR participation)

AND

("mental health*" OR "mental illness*" OR "psychiatric illness*")

AND

(power OR “interpersonal control” OR “social power” OR “psychological power” OR “professional power”)
